# Ungated cine first-pass CMR for concurrent imaging of myocardial perfusion defects and wall motion abnormalities

**DOI:** 10.1186/1532-429X-15-S1-O1

**Published:** 2013-01-30

**Authors:** Behzad Sharif, Rohan Dharmakumar, Reza Arsanjani, Louise E Thomson, Noel Bairey Merz, Daniel S Berman, Debiao Li

**Affiliations:** 1 Biomedical Imaging Research Institute, Cedars-Sinai Medical Center, Los Angeles, CA, USA; 2 Heart Institute, Cedars-Sinai Medical Center, Los Angeles, CA, USA

## Background

Combined assessment of wall motion from cine imaging and perfusion defects from first-pass perfusion (FPP) imaging has been shown to have a high diagnostic performance for detection of acute ischemia. In this setting, a single ungated CMR scan capable of simultaneously capturing perfusion deficits and wall motion abnormality can be useful for rapid diagnosis of ongoing acute ischemia. We propose an accelerated FPP technique with ungated continuous acquisition capable of generating cardiac-phase resolved FPP images thereby enabling concurrent imaging of wall motion and perfusion deficits.

## Methods

FPP imaging without magnetization preparation using a steady state acquisition has been described before [1] and seen recent interest [2,3], wherein the focus has been on acquiring one image during the quiescent phase. Canines with reversible ischemia were studied (N=5; >90% LAD stenosis for 4, no stenosis for 1). Resting FPP data was acquired on a 3T scanner (Siemens Verio) using an ungated RF-spoiled GRE sequence with continuous golden-angle radial acquisition of 1 slice (called "cine FPP"; resolution: 1.5x1.5x6 mm, 30 sec scan, 13000 projections, flip=14°). All scans were performed 7±2 minute post occlusion and the mean heart rate (HR) was 98 bpm. Image reconstruction was performed using a regularized iterative SENSE scheme (temporal resolution: 61 ms). For comparison, a conventional gated SR-prepared FLASH "standard FPP" scan was also acquired.

## Results

**Figure 1:**
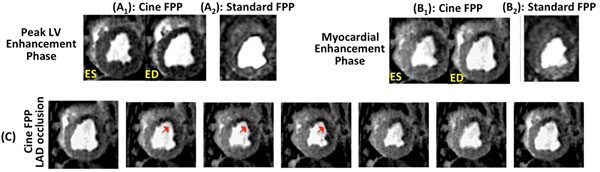
Panels **(A_1_)** and **(B_1_)** show cine FPP images (systolic and diastolic phases) in two different contrast enhancement phases: peak LV bloodpool enhancement; and myocardial enhancement. Panels **(A_2_)** and **(B_2_)** show the corresponding images from the standard FPP scan. Row **(C)** shows 7 frames (frame rate: 16 frames/s) from one heartbeat of the ungated cine FPP images during myocardial enhancement. Arrows point to the hypokinetic wall.

**Figure 2:**
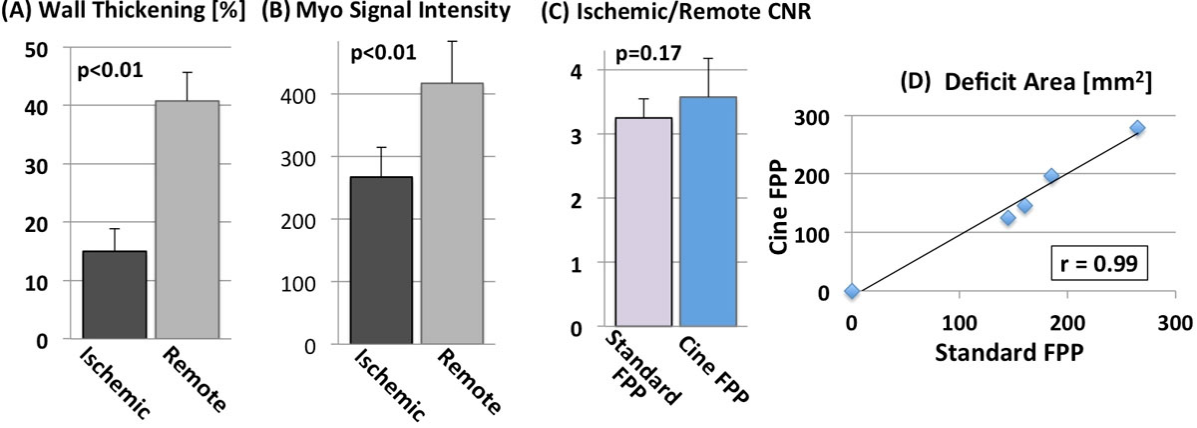
Panels **(A)** and **(B)** show the results of wall motion (systolic wall thickening as a percentage of diastolic thickness) and myocardial signal intensity from the cine FPP images in the 4 ischemic dogs. For **(A)**, consecutive frames from peak LV enhancement and for **(B)** a diastolic frame during myocardial enhancement phase were analyzed. Panel **(C)** compares the ischemic-to-remote myocardial image contrast (for the 4 ischemic dogs) of the cine FPP images to standard FPP (SR-prepared ECG-gated FLASH), which shows that cine FPP has slightly higher CNR (3.6 vs. 3.3; statistically insignificant). Finally, **(D)** compares the detected deficit area (in mm^2^) between cine and standard FPP in all 5 studied dogs (1 with no occlusion), which shows a very good correlation (r=0.99).

The top row of Fig. 1 shows cine FPP images (systolic/diastolic) in peak LV and myocardial enhancement phases, along with the corresponding images from the standard FPP scan. Fig. 1(C) shows 7 frames (16 frames/s) from the cine FPP in the myocardial enhancement phase. Arrows point to hypokinesia. Figs. 2(A)-(B) show the result of wall motion and myocardial signal intensity analysis from the cine FPP images. Fig. 2(C) compares the myocardial contrast properties of the cine FPP images to standard FPP, showing similar ischemic-to-remote CNR. Finally, 2(D) compares the detected deficit area between cine and standard FPP, showing a positive correlation (r=0.99).

## Conclusions

We have demonstrated, for the first time, the feasibility and effectiveness of ungated cardiac-phase resolved (cine) FPP imaging for concurrent imaging of myocardial wall motion and perfusion in an animal model with flow-limiting stenosis. The presented method may improve the feasibility of detecting acute myocardial ischemia using CMR because of its reduced scan time (single scan for both cine and FPP) and reduced complexity (no cardiac gating). It may also enhance the accuracy and speed of diagnosis by virtue of concurrent (inherently fused) imaging of wall motion and FPP. The presented results demonstrate that the method is capable of imaging at high HRs with high spatial and sufficient temporal resolution. While the current method is limited to imaging a single slice during a breathhold, it can potentially be extended to 3D through spatio-temporal acceleration.

## Funding

Grant sponsors: American Heart Association Postdoctoral Fellowship Award 11POST7390063; National Institutes of Health grants nos. NHLBI HL38698, HL091989.

## References

[B1] Judd MRM 1995 34

[B2] DiBella MRM 2012 67

[B3] Giri ISMRM 2012 3890

